# From bench to bytes: a practical guide to RNA sequencing data analysis

**DOI:** 10.3389/fgene.2025.1697922

**Published:** 2025-10-28

**Authors:** Prabin Dawadi, Bivek Pokharel, Anita Shrestha, Dikshya Niraula, Afifa Naeem, Sayaka Miura, Mishal Roy, Saroj Nepal

**Affiliations:** Department of Biology, University of Mississippi, Oxford, MS, United States

**Keywords:** Beginner’s guide, bioinformatics, DESeq2, gene expression analysis, RNA-Seq

## Abstract

RNA sequencing (RNA-Seq) is a high-throughput sequencing approach that enables comprehensive quantification of transcriptomes at a genome-wide scale. As a result, RNA-Seq has become a routine component of molecular biology research, and more researchers are now expected to analyze RNA-Seq data as part of their projects. However, unlike the largely experimental nature of benchwork, RNA-Seq analysis demands proficiency with computational and statistical approaches to manage technical issues and large data sizes. Although numerous manuals and reviews on RNA-Seq data analysis are available, many are either highly specialized, fragmented, or overly superficial, leaving beginners to use tools without understanding the underlying principles. To address this gap, we provide a decision-oriented guide tailored for molecular biologists encountering RNA-Seq analysis for the first time. This review is designed for readers to enable to decide which tools and statistical approaches to use based on their data, goals, and constraints. We aim to equip beginners with the knowledge required to perform RNA-Seq analysis rigorously and with confidence.

## 1 Introduction

RNA-Seq is a powerful high-throughput technology that has revolutionized the study of transcriptomics by enabling genome-wide quantification of RNA abundance. Compared to earlier methods (e.g., microarrays), it offers more comprehensive coverage of the transcriptome, finer resolution of dynamic expression changes, and improved signal accuracy with lower background noise, making it the preferred approach for gene expression analysis in modern molecular biology and medicine (Hrdlickova et al., 2017; Koch et al., 2018; Kukurba and Montgomery, 2015). It enables researchers to address a diverse array of biological questions, spanning from disease biomarker discovery and drug identification to advancing the understanding of developmental biology, host-pathogen dynamics, and responses to environmental stimuli (Berger et al., 2010; Navin, 2014). RNA-Seq technologies are expected to continue advancing, with their application expanding even further in the future.

**FIGURE 1:**
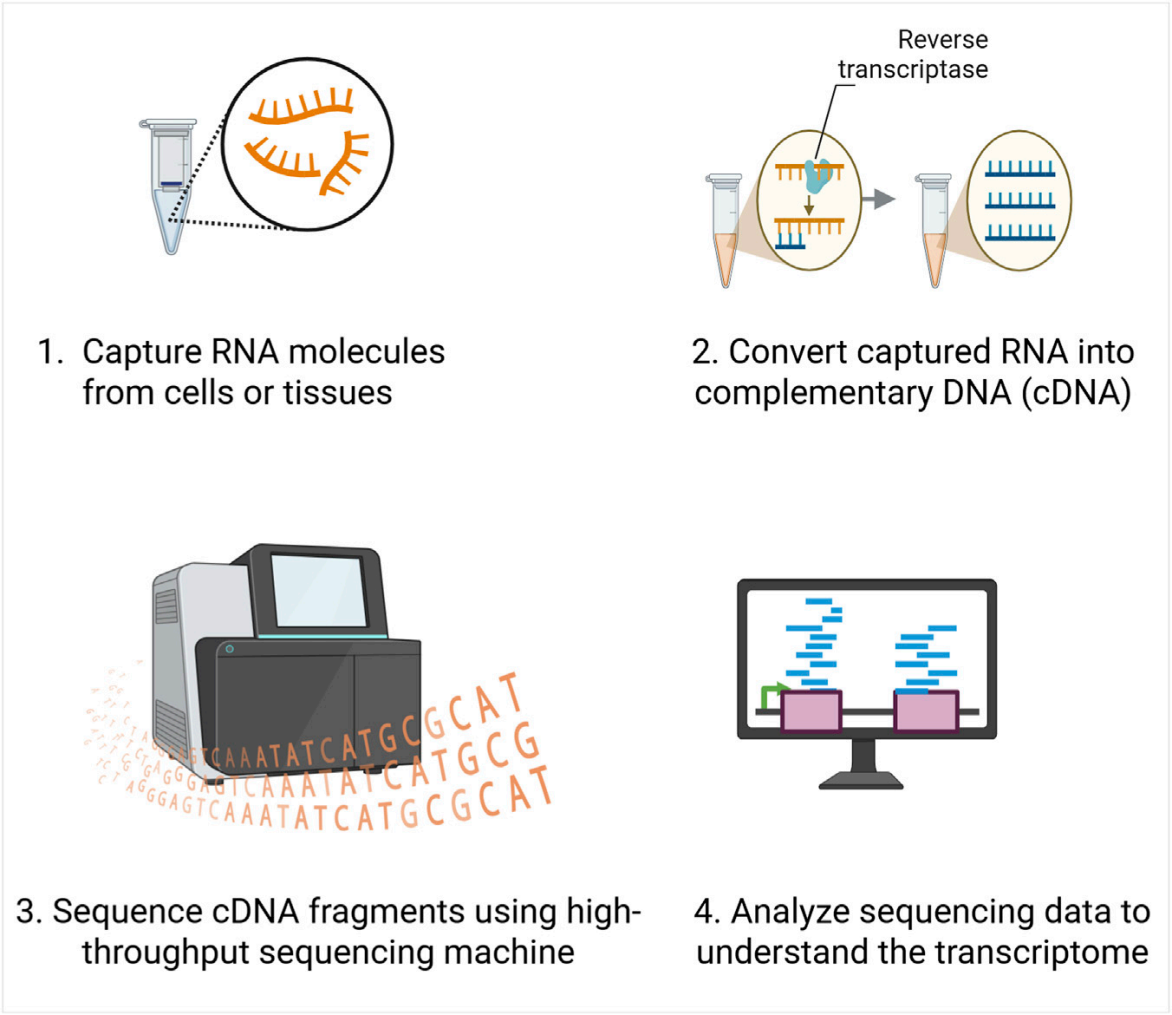
Overview of RNA sequencing workflow. RNA molecules are first extracted from cells or tissues, then converted into complementary DNA (cDNA) using reverse transcriptase. The resulting cDNA fragments are sequenced using high-throughput sequencing platforms, and the data are computationally analyzed.

RNA-Seq works by first isolating the RNA molecules from cells or tissues and then converting them into complementary DNA (cDNA), because DNA is more stable and easier to handle in downstream workflows (Figure 1). These cDNA fragments are then sequenced using high-throughput sequencers, which read out millions of short sequences (reads) at once. Thus, each read represents a fragment of an RNA molecule that was present in the sample at the time of sequencing. Collectively, these reads capture the transcriptome, reflecting both the identity and abundance of expressed genes.

Since RNA-Seq data consists of sequenced reads, its data structure poses significant challenges for beginners. For example, RNA-Seq data is typically stored in text-based formats such as FASTQ (raw reads with quality scores), SAM/BAM (aligned reads), or count matrices (summarized expression levels). As a result, novices often struggle both with handling RNA-Seq data stored in specialized formats and with understanding the unique characteristics of RNA-Seq data when making biological inferences (Conesa et al., 2016; Love et al., 2014; Trapnell et al., 2012).

With varying audiences and objectives, several foundational reviews and guides on RNA-Seq analysis have made significant contributions to the field. For example, Conesa et al. (2016) provide comprehensive coverage of best practices across diverse applications, which is especially valuable for readers seeking detailed methodological insights. The tool-oriented review of differential gene expression (DGE) analysis by Costa-Silva et al. (2017) helps readers identify suitable software options. Likewise, the Bioconductor guide by Love et al. (2015) offers detailed instructions for statistical testing and visualization in R, while Koch et al. (2018) present broad checkpoints to support researchers in evaluating their workflows. Collectively, these resources establish a strong foundation for RNA-Seq analysis.

Our review goes beyond listing tools or workflows. We provide a decision-oriented guide that organizes available methods around the key choices researchers must make, such as computational tools and statistical techniques. By clarifying the assumptions behind statistical models and illustrating common pitfalls, we aim to fill a gap between descriptive reviews and practical decision-making. This perspective actively guides researchers particularly newcomers toward their DGE analyses with confidence.

## 2 Preprocessing RNA-Seq data

**FIGURE 2:**
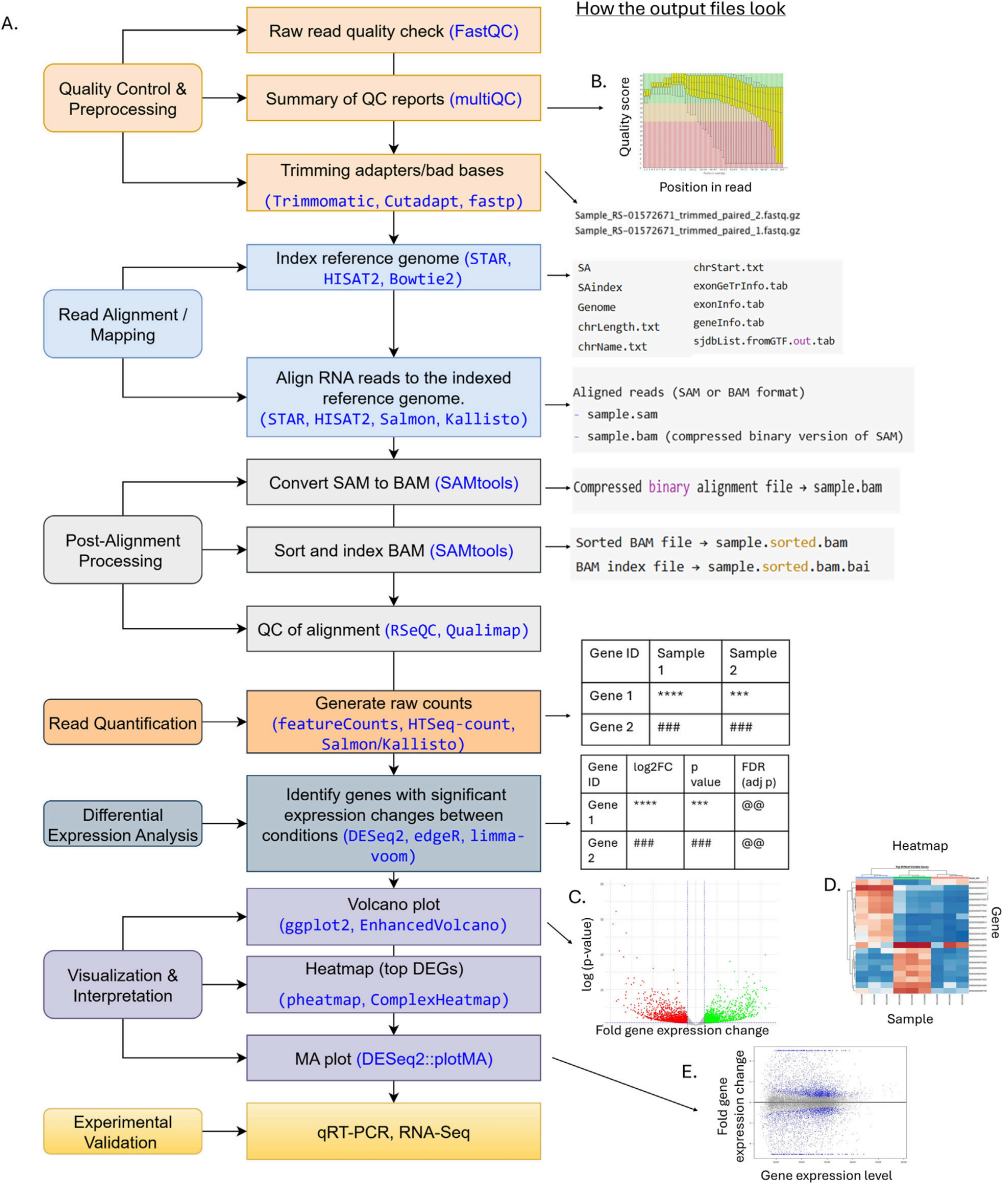
Flowchart illustrating an RNA-Seq analysis pipeline, from raw FASTQ files to functional interpretation. **(A)** Steps and tools. Each step includes key steps shown on the left and commonly used tools (in blue) and corresponding outputs (files, tables, or figures) displayed on the right next to the analysis. **(B)** Example of low-quality data using FastQC. The red and blue line is the median and mean of quality score at a given position of a read. The yellow box represents the inter-quartile range (25%–75%), and the upper and lower whiskers represent the 10% and 90% points. The higher the quality score is the better the base call. The background of the graph (green, orange, and red) indicates very good, reasonable, and poor quality calls, respectively. **(C)** Volcano plot. **(D)** Heatmap. **(E)** MA plot.

Figure 2 shows steps in RNA-Seq data analysis. The analysis begins with cleaning sequenced data and counting how many sequencing reads are mapped to each gene or transcript (Conesa et al., 2016). The detailed protocol and usages of required computational tools for this preprocessing step are summarized in Shouib et al. (2025).

**FIGURE 3:**
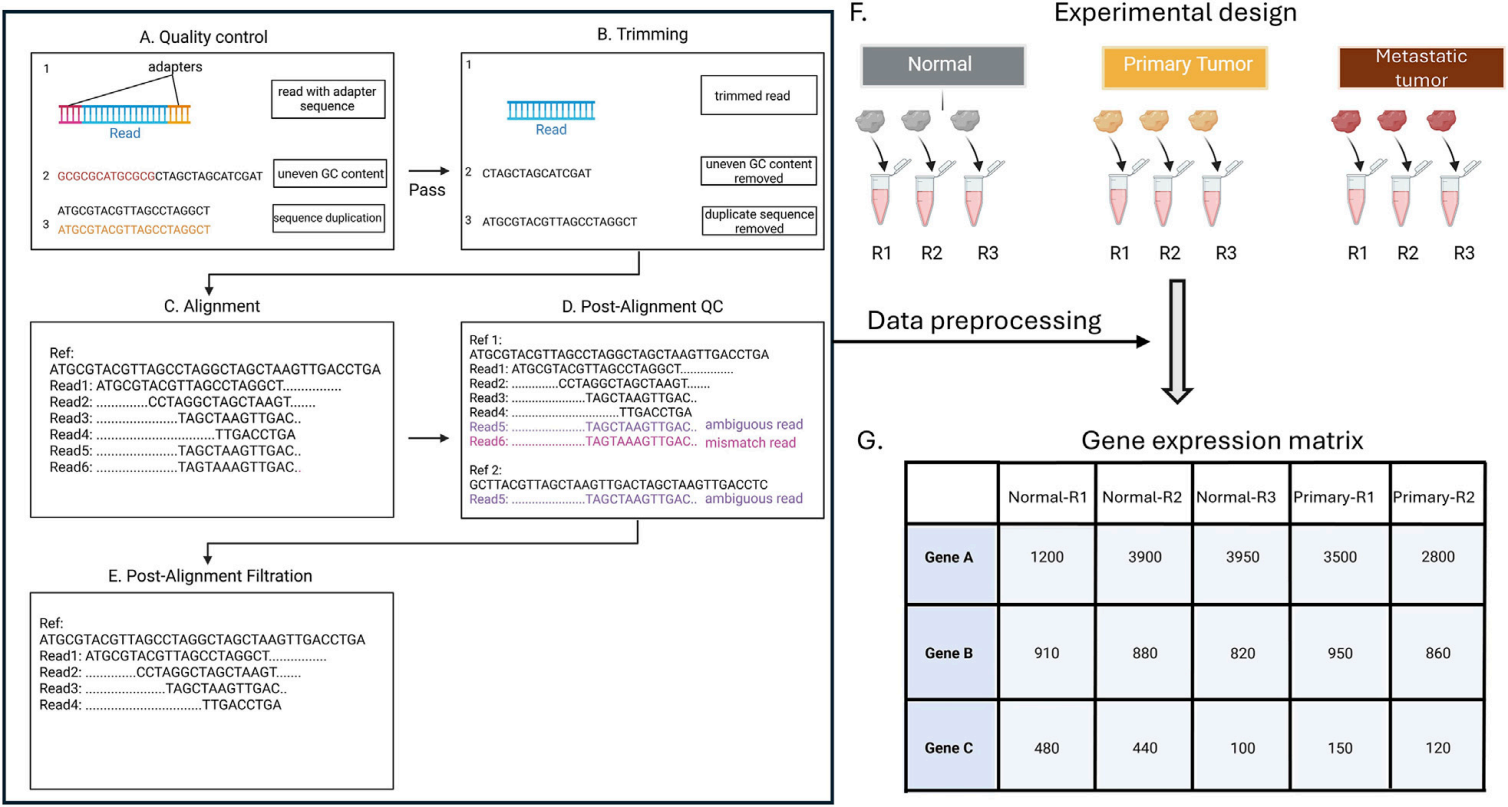
Quality control (QC) of RNA-Seq data. Sequencing reads undergo QC to identify adapter contamination, GC bias, and duplicate sequences **(A)**, followed by trimming to remove artifacts **(B)**. Reads are then mapped to reference transcripts (Ref) **(C)**, and post-alignment QC is performed to detect mismatches and ambiguous mappings **(D)**. After post-alignment filtration, high-confidence reads are retained for quantification **(E)**, generating gene-level counts across experimental conditions for downstream analysis **(F,G)**. In the experimental design for differential expression analysis. each of the example groups (healthy, primary tumor, and metastatic tumor) has three replicates (R1, R2, R3).

Briefly, the first quality control (QC) step identifies potential technical errors, such as leftover adapter sequences, unusual base composition (technical sequences), or duplicated reads (Figure 3A). Tools like FastQC or multiQC are commonly used (Ewels et al., 2016; Wingett and Andrews, 2018). It is critical to review QC reports (Figure 2B) and to ensure that errors are removed without cutting too many good reads during trimming, as over-trimming reduces data and weakens analysis.

After QC is passed, the next step is read trimming, which cleans the data by removing low-quality parts of the reads and leftover adapter sequences that can interfere with accurate mapping (Figure 3B) (Bolger et al., 2014). Tools like Trimmomatic, Cutadapt, or fastp are commonly used for this step (Chen et al., 2018; Martin, 2011).

Once the reads are cleaned, they are aligned (mapped) to a reference transcriptome using software such as STAR, HISAT2, or TopHat2 (Figure 3C) (Dobin et al., 2013; D. Kim et al., 2015; Trapnell et al., 2009). This step identifies which genes or transcripts are being expressed in the samples (Engström et al., 2013). An alternative is pseudo-alignment with Kallisto or Salmon, which estimate transcript abundances without full base-by-base alignment (Bray et al., 2016). These methods are faster and use less memory, making them well suited for large datasets. Both Kallisto and Salmon incorporate statistical models (bootstrapping) to improve accuracy.

After alignment, post-alignment QC is performed by removing reads that are poorly aligned or mapped to multiple locations, using tools like SAMtools, Qualimap, or Picard (Figure 3D) (Li et al., 2009; Okonechnikov et al., 2016). This step is essential because incorrectly mapped reads can artificially inflate read counts. As a result, gene expression levels may appear higher than they truly are, which can distort comparisons of expression between genes in downstream analyses.

The final step is read quantification, where the number of reads mapped to each gene is counted (Figure 3E) (Liao et al., 2014). Tools like feature Counts or HTSeq-count perform this counting, producing a raw count matrix. This matrix summarizes how many reads were observed for each gene in each sample, where a larger number of reads indicates higher gene expression (Anders et al., 2015).

## 3 Experimental design and normalization techniques

A popular usage of RNA-Seq is the identification of differentially expressed genes between conditions, such as treated versus control groups (Figure 3F). The reliability of DGE analysis depends strongly on thoughtful experimental design, particularly with respect to biological replicates and sequencing depth.

With only two replicates, DGE analysis is technically possible, but the ability to estimate variability and control false discovery rates is greatly reduced. A single replicate per condition, although occasionally used in exploratory work, does not allow for robust statistical inference and should be avoided for hypothesis-driven experiments. While three replicates per condition is often considered the minimum standard in RNA-seq studies, this number is not universally sufficient. In general, increasing the number of replicates improves power to detect true differences in gene expression, especially when biological variability within groups is high (Liu et al., 2014; Schurch et al., 2016).

Sequencing depth is another critical parameter. Deeper sequencing captures more reads per gene, increasing sensitivity to detect lowly expressed transcripts. For standard DGE analysis, ∼20–30 million reads per sample is often sufficient (Conesa et al., 2016; The Encode Project Consortium, 2011). Estimating depth requirements prior to sequencing can be guided by pilot experiments, existing datasets in similar systems, or tools that model detection power as a function of read count and expression distribution, e.g., Scotty (Busby et al., 2013).

**FIGURE 4:**
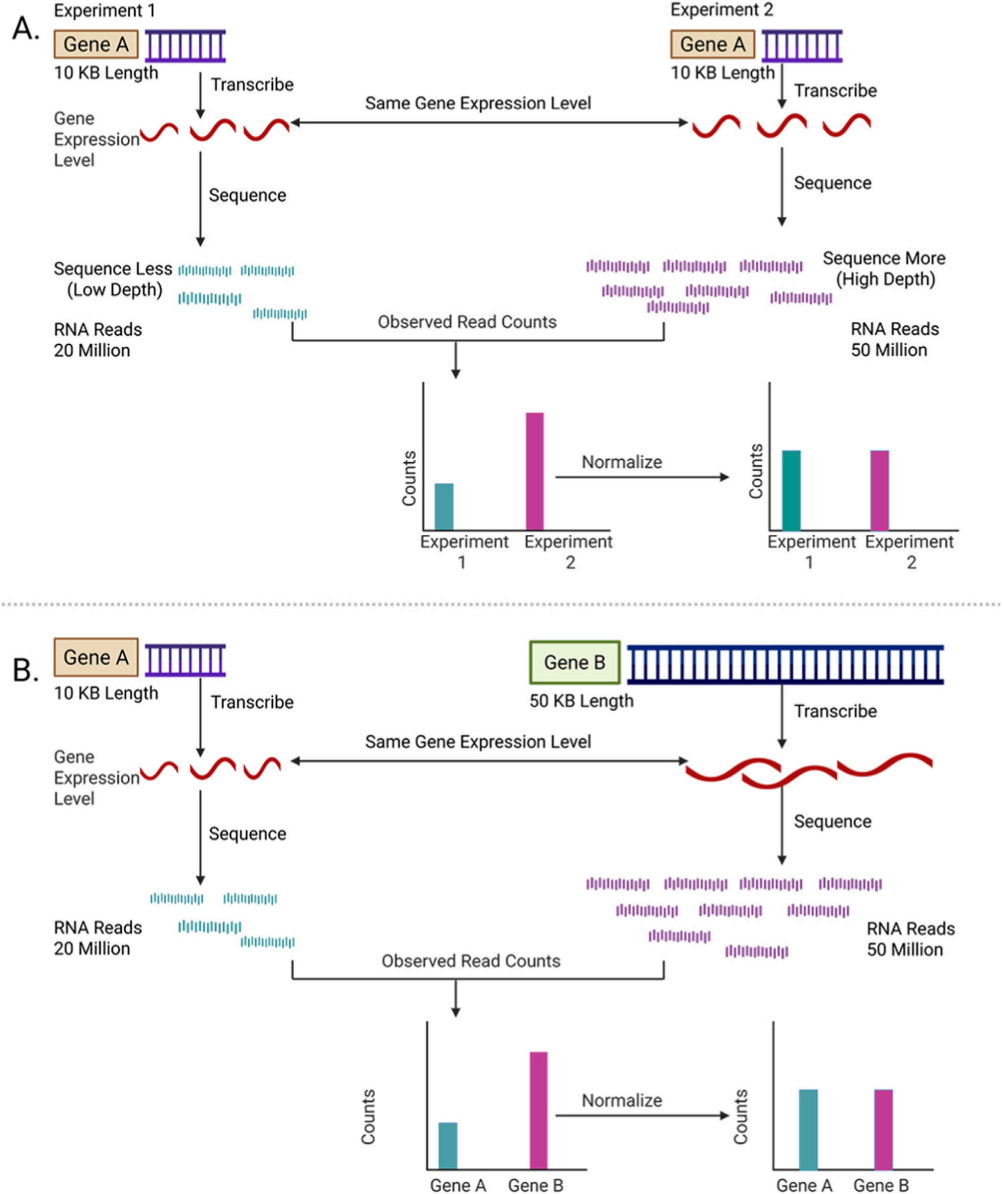
Schematic overview of normalization in RNA-Seq. **(A)** Normalization across experiments. Raw read counts for the same gene (Gene A) differ due to sequencing depth or technical variation between experiments. Normalization adjusts these differences to reflect true expression levels. **(B)** Normalization across genes. Genes of different lengths (Gene A vs. Gene B) yield different read counts even at similar expression levels. Normalization corrects for gene length bias, enabling fair comparison across genes.

The raw counts in the gene expression matrix generated in the previous section (Figure 3G) cannot be directly comparable between samples because the number of reads mapped to a gene depends not only on its expression level but also on the total number of sequencing reads obtained for that sample, called the sequencing depth (Han and Men, 2018; Risso et al., 2014). Samples with more total reads will naturally have higher counts, even if the genes are expressed at the same level (Figure 4A). “Normalization” adjusts these counts mathematically to remove such biases (Zyprych-Walczak et al., 2015).

There are various normalization techniques (Table 1). A simple normalization method is Counts per Million (CPM), where raw read counts for each gene are divided by the total number of reads in the library (sequencing depth), then multiplied by one million. Thus, CPM assumes that all samples are comparable if they were sequenced to the same depth. However, this assumption often fails in real experiments. For example, if a few genes are extremely highly expressed in one sample, they consume a large fraction of the total reads. This creates a misleading picture when comparing across samples.

**TABLE 1:** Summary of normalization techniques.

Methods	Sequencing depth correction	Gene length correction	Library composition correction	Suitable for DE analysis	Notes
CPM (Counts per Million)	Yes	No	No	No	Simple scaling by total reads; affected by highly expressed genes
RPKM / FPKM (Reads/fragments per Kilobase of Transcript, per Million Mapped Reads)	Yes	Yes	No	No	Adjusts for gene length; still affected by library composition
TPM (Transcripts per Million)	Yes	Yes	Partial	No	Scales sample to constant total (1M), reducing composition bias; good for visualization and cross-sample comparison
median-of-ratios	Yes	No	Yes	Yes	Implemented in DESeq2; affected by expression shifts
TMM (Trimmed Mean of M-values)	Yes	No	Yes	Yes	Implemented in edgeR; affected by over-trimming genes

More advanced methods are implemented in DGE analysis tools (e.g., DESeq2 and edgeR), which can correct for differences in library composition. For example, DESeq2 uses median-of-ratios normalization, which uses a size factor to adjust for sequencing depth. It first calculates a reference expression level for each gene, i. e., average across all samples. Each sample’s gene expression is then compared to this reference to get ratios, and the median ratio is defined as the “size factor.” Raw counts are divided by this factor to make samples comparable (Hafemeister and Satija, 2019; Love et al., 2014).

A potential issue of this median-of-ratios normalization is the assumption that most genes have similar expression across samples. Thus, when a large number of genes have different expression levels between samples, the normalized values may be misleading (Dillies et al., 2013).

The edgeR employs another method, Trimmed Mean of M-values (TMM), where genes that are extremely highly or lowly expressed, or strongly differentially expressed, are excluded (this is the “trimming” step). The remaining set of genes is used to calculate an average log-fold change (the “mean of M-values”), which is used as a scaling factor (Robinson et al., 2010; Singh et al., 2024).

TMM assumes that the remaining genes reflect true depth differences (Robinson and Oshlack, 2010). Thus, a pitfall is that it may over-trim, leading to incorrect scaling.

The code below demonstrates how to set up and perform normalization using DESeq2, a popular R package for DGE analysis. The first line installs the package, and the second line loads it into the R session so its functions can be used. The next line creates a variable called “biopsy_site,” which records the experimental groups for each sample (here: “N” = normal, “P” = primary tumor, “M” = metastatic tumor). These labels tell DESeq2 how the samples are organized. The DESeqDataSetFromMatrix function then combines the raw count data (counts) with the sample information to create a special object (dds) that DESeq2 can work with. After this, estimateSizeFactors calculates scaling factors to account for differences in sequencing depth across samples. Finally, the counts function with normalized = TRUE produces a table of normalized counts.

*BiocManager::install (“DESeq2”) #install the package*
*library (DESeq2) #load the package*
*#Format data*
*biopsy_site < - factor (c (“N”, “N”, “N”, “P”, “P”, “P”, “M”, “M”, “M”))*
*dds < - DESeqDataSetFromMatrix (counts, DataFrame (biopsy_site), ∼ biopsy_site)*
*dds < -estimateSizeFactors (dds) #Add the scaling factor into dds*
*dds_normalized < - counts (dds, normalized = TRUE) #Generate normalized counts*

These sequencing depth adjustments attempt to ensure that read counts are comparable across samples, but they do not account for differences in gene length. Longer genes naturally generate more reads than shorter genes, even if both are expressed at the same level per base (Figure 4B). Methods such as RPKM (reads per kilobase of transcript, per million mapped reads) additionally normalize the read counts by gene length. Thus, these methods allow comparisons of expression levels between genes within a single sample. However, RPKM is not recommendable to use for between-sample comparisons, because they do not fully correct for the composition bias (just like CPM).

Transcripts per Million (TPM) quantifies gene expression in a way that allows comparisons both between genes within a sample and for the same gene across samples. TPM first adjusts raw read counts for gene length. The length-adjusted counts are then scaled so that the total expression across all genes in a sample sums to a constant (one million). However, TPM does not model biological or technical variability (Pachter, 2011). TPM is therefore primarily used for visualization, reporting, and exploratory cross-sample comparisons rather than for rigorous statistical testing.

## 4 Data validation and scaling techniques

Before identifying differentially expressed genes, it is important to first validate the data. In an experiment, we expect that biological replicates within the same group will show similar gene expression patterns, while samples from different groups should be more distinct. Checking whether this expectation holds true is a key step in quality assessment.

**FIGURE 5:**
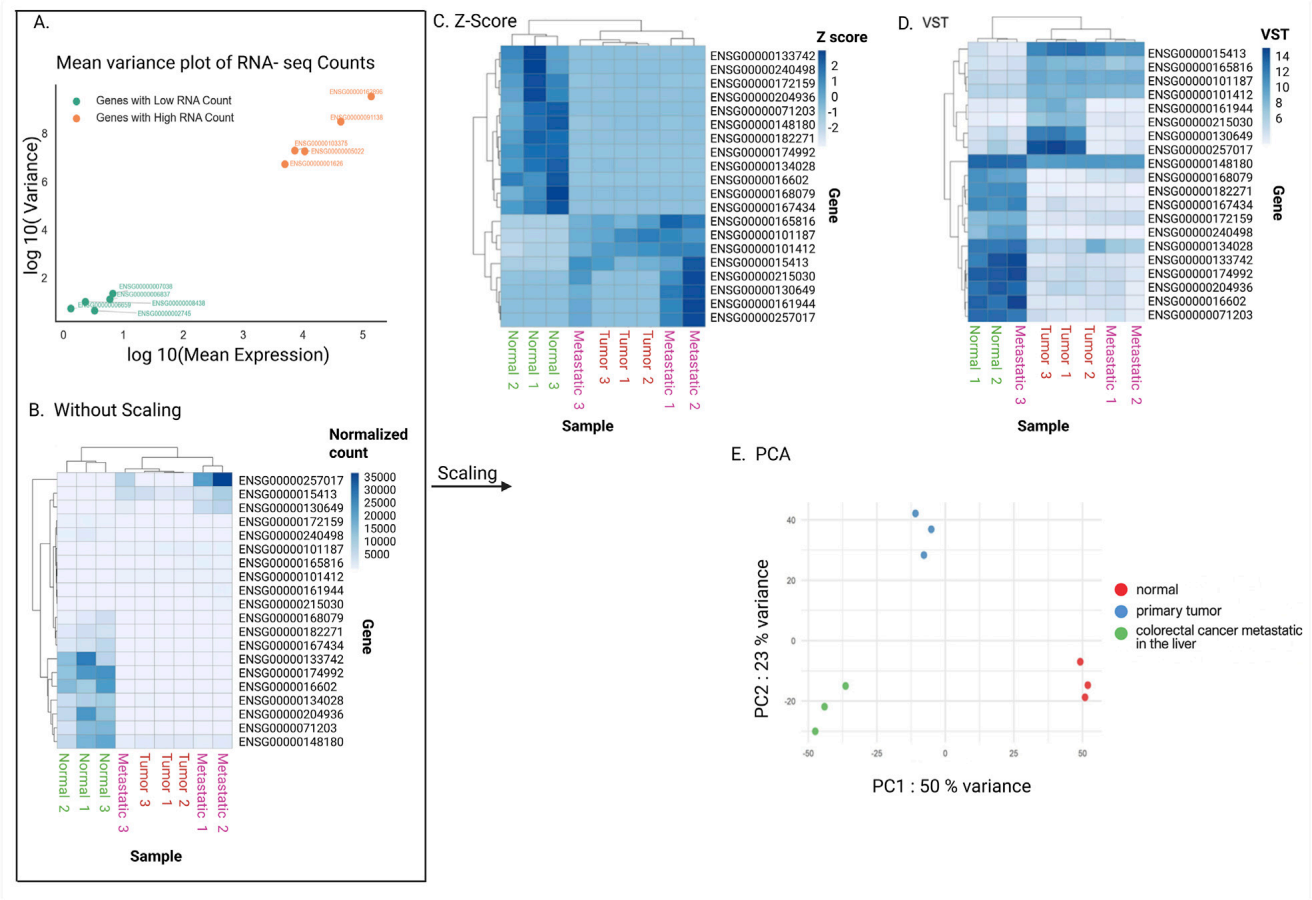
Scaling data. **(A)** Mean and variance of gene expression level. Variance is larger for larger mean in a count data. To compare expression variability, five genes with low RNA counts and five genes with high RNA counts were randomly selected from the “Normal” biological sample. **(B–D)** Heatmaps without scaling gene expression levels **(B)**, with z-score **(C)**, and with VST (Variance Stabilizing Transformation) **(D)**. The top 20 detected genes with the best adjusted p-values in the differential expression gene analysis were used. **(E)** Principal component analysis (PCA). The top 1,000 most variable genes were used. The x-axis represents PC1, accounting for 50% of the variance, while the y-axis represents PC2, accounting for 23% of the variance. Data points are color-coded: red for normal tissue, blue for primary tumor, and green for metastatic tumor.

To do this, we often compare overall gene expression patterns between samples. However, using normalized counts directly can be misleading. This is because variance in normalized read count depends strongly on expression level, where highly expressed genes naturally show more variability than lowly expressed ones (Figure 5A). This variability does not reflect true biology. At the same time, differences between lowly expressed genes tend to be compressed compared to those of highly expressed genes (Figure 5B). If we treat all genes equally without accounting for this, the similarity between samples will be disproportionately driven by a small set of highly expressed genes, while the variable variance across expression levels can distort the overall picture.

To address this, the data are further transformed, or “scaled,” so that differences between genes are placed on a more comparable footing. The most straightforward way to reduce differences in variance across genes is a log transformation, typically log_2_ (counts +1) or log_2_ (counts per million +1). This compresses large values and stretches out small ones, which helps make genes with different expression levels more comparable. For example, a gene with 1,000 counts and another with 10 counts become closer in scale after the log is applied.

The underlying assumption is that a log scale can stabilize variance well enough for exploratory analyses. However, this approach has important limitations. Very low counts can be unstable because a difference of just one read leads to a large change after logging, while very high counts may still dominate overall patterns. This means log transformation alone may exaggerate noise in lowly expressed genes and not fully correct the dependence of variance on mean expression. Despite these issues, log transformation remains useful in situations when a quick, computationally simple method is needed for exploratory visualization.

A more sophisticated method is z-score scaling (Eisen et al., 1998). Here, each gene’s expression values are centered around its mean and scaled by its variability across samples. In practical terms, this shows whether a gene is expressed higher or lower than its average in a given sample, rather than focusing on its absolute expression level (Figure 5C). The assumption behind z-scoring is that each gene’s variability is meaningful and comparable across the dataset. However, this can also be a limitation: if a gene has very low counts or unreliable measurements, the z-score may exaggerate noise. Additionally, it is important to note that z-scores remove information about absolute expression levels, which may be biologically important in some contexts.

Another method is the variance stabilizing transformation (VST), implemented in DESeq2. VST uses a mathematical model with a smooth relationship between variance and mean expression estimated using all genes (Figure 5D). The main assumption of VST is that most genes are not strongly differentially expressed, which allows the method to model a smooth relationship between mean expression and variance across the transcriptome. However, this assumption can be violated, in experiments where a large fraction of genes are globally up- or downregulated (such as strong perturbations, cross-tissue comparisons, or conditions that trigger widespread transcriptional reprogramming). In such cases, the variance trend may be biased, and VST can over- or under-correct variability for some genes, leading to noisier exploratory plots.

Another strategy is the regularized log transformation (rlog), which is also available in DESeq2. In rlog, low-count genes are adjusted more strongly to reduce the noise that comes from sampling variation, while high-count genes are treated more like a standard log transformation. Thus, a limitation is that if a dataset has many genes with true biological variability at low counts, rlog might shrink too aggressively, dampening real biological differences. Also, it requires a higher computational cost, i.e., rlog is slower and more memory-intensive than VST.

The code below transforms (scales) the normalized counts using DESeq2. The functions vst (dds) perform Variance Stabilizing Transformation and store the counts in the objects (vsd).

*vsd < - vst (dds, blind = FALSE)*
*norm_counts < - assay (vsd)*

These scaling techniques are summarized in Table 2. Overall, these scaled values allow overall gene expression patterns to be compared more fairly between samples, making it easier to identify consistent replicates, detect outliers, assess group separation, and reveal potential technical artifacts. Principal Component Analysis (PCA) is a widely used method for this purpose. PCA reduces the complexity of large datasets by summarizing overall gene expression patterns into a few “principal components,” which capture the most variation between samples (Maćkiewicz and Ratajczak, 1993). In a resulting PCA plot, the x-axis (PC1) and y-axis (PC2) represent the main patterns of variation in the dataset (Holland, 2008). For example, in Figure 5E, PC1 captures 50% of the differences between samples, meaning it reflects the largest source of variation, while PC2 captures 23% of the differences, representing the second most important source. Together, these two axes summarize 73% of the overall variation, providing a clear picture of how samples relate to each other based on their gene expression profiles.

**TABLE 2:** Summary of scaling (transformation) techniques.

Transformation	Assumption	Strengths	Limitations	Best use case
Log (e.g., log₂(counts+1))	Variance can be stabilized by simple compression of large values	Simple and intuitive	Unstable for very low counts; high-expression genes may still dominate	Quick exploratory plots
Z-score	Each gene’s variability is meaningful across samples	Highlights relative differences within genes	Removes absolute expression scale; exaggerates noise	Quick exploratory plots
VST (Variance Stabilizing Transformation, DESeq2)	Most genes are not strongly differentially expressed	Handles low counts better than simple log	Assumption may break if most genes shift	Clustering
rlog (Regularized log, DESeq2)	Variation in low-counts is noise	Reduces noise from low-count genes	Dampen real biological differences for lowly-expressed genes; Computationally intensive	Small datasets with strong expression differences

In a PCA plot, each point represents a sample, and the distance between points reflects how similar or different their overall gene expression profiles are. Samples that cluster closely together indicate consistent replicates, while points that are distant from their group may indicate outliers or technical issues.

PCA is typically performed using a subset of genes, i.e., highly variable genes. However, including too many low-variance genes may add noise, while selecting only high-variance genes may bias the principal components toward specific features. Another limitation is that PCA is a linear method that captures the largest sources of variation, providing a global overview of sample relationships, but it may miss subtle local patterns.

The R function below produces a PCA plot using the VST object (see above code). The argument ntop = 1,000 tells DESeq2 to use the 1,000 most variable genes when calculating the PCA.

*pcaData < - plotPCA(vsd, intgroup = “biopsy_site”, ntop = 1,000, returnData = TRUE)*

## 5 Differential gene expression (DGE) analysis

After the data is validated, differentially expressed genes between defined groups can be identified. The goal is to compare gene expression patterns between the samples and determine whether observed differences in normalized read counts between groups reflect true biological changes or simply random noise.

Specialized statistics are necessary because normalized read counts vary widely across genes: some genes are highly expressed and may have thousands of reads, while others are lowly expressed and may have only a few (Figure 5A). Importantly, the variance of counts is not constant, i.e., highly expressed genes tend to show greater variability across samples than lowly expressed genes. This violates the assumptions of simple statistical tests, such as t-tests, which assume normally distributed data with roughly equal variance across all observations.

There are various statistical methods to consider this difference in the variability of read counts (Table 3). For example, DESeq2 assumes that most genes are not strongly differentially expressed and models raw read counts with a negative binomial distribution (Love et al., 2014). To stabilize variance estimates, it borrows information across all genes, which is particularly useful for genes with low read counts where variability is high. This makes DESeq2 robust for experiments with a moderate number of replicates. However, if a large proportion of genes shift in the same direction—such as in global transcriptional changes—this assumption may break down, leading to biased variance estimates and potentially inflated false positives.

**TABLE 3:** Summary of differential expression analysis techniques.

Method	Normalization	Assumption	Statistical approach	Strengths	Weaknesses	Best use case
DESeq2	(median-of-ratios)	Most genes are not strongly differentially expressed; counts follow negative binomial	Robust variance estimation by borrowing information across genes	Handles low-count genes well	Sensitive to global shifts	Moderate replicates; when stable variance estimation is important
edgeR	(TMM)	dispersions vary across genes; counts follow negative binomial	Empirical Bayes shrinkage for dispersion	Effective for small sample sizes	Sensitive to outliers and very low-count genes	Small experiments; accurate dispersion estimation needed
limma-voom	log-CPM	Variance depends on mean	Models with mean–variance relationship	Computationally efficient	Less accurate for very low-count genes	Large datasets

edgeR also uses a negative binomial framework, but it differs in how it estimates gene-specific dispersion. By applying an empirical Bayes approach, edgeR shrinks gene-wise dispersions toward a common trend, allowing more stable inference even with very few replicates (Robinson and Oshlack, 2010). This makes it powerful in small-sample studies. However, its main limitation is sensitivity to extreme outliers and very low-count genes, which can distort dispersion estimates.

Note that DESeq2 and edgeR do not use normalized read counts directly in their statistical models. Instead, they incorporate normalization factors (size factors in DESeq2, scaling factors in edgeR) into the model as offsets. Thus, the DE testing itself is performed on the raw counts with normalization applied internally.

On the other hand, limma-voom adopts a fundamentally different strategy: rather than modeling counts directly, it transforms them to log-CPM values and estimates the mean–variance relationship to assign precision weights (Law et al., 2014). This allows limma’s linear modeling framework, originally developed for microarray data, to be applied efficiently to RNA-Seq. The method is fast and performs well with large sample sizes. Its limitation lies in handling very low-count genes, where log-transformation can exaggerate noise.

Lastly, these approaches analyze gene expression patterns for each gene to identify which genes are expressed differently between the groups being studied. However, because RNA-Seq experiments often test thousands of genes at once, some genes will appear to be “significant” just by random chance, even if they are not truly different. To correct for this problem, the raw p-values from the statistical tests are adjusted using methods such as the Benjamini–Hochberg’s approach using the false discovery rate (FDR) (Benjamini and Hochberg, 1995). The resulting adjusted p-values (often called q-values) provide a more reliable measure of significance, which is used for deriving biological inferences.

**FIGURE 6:**
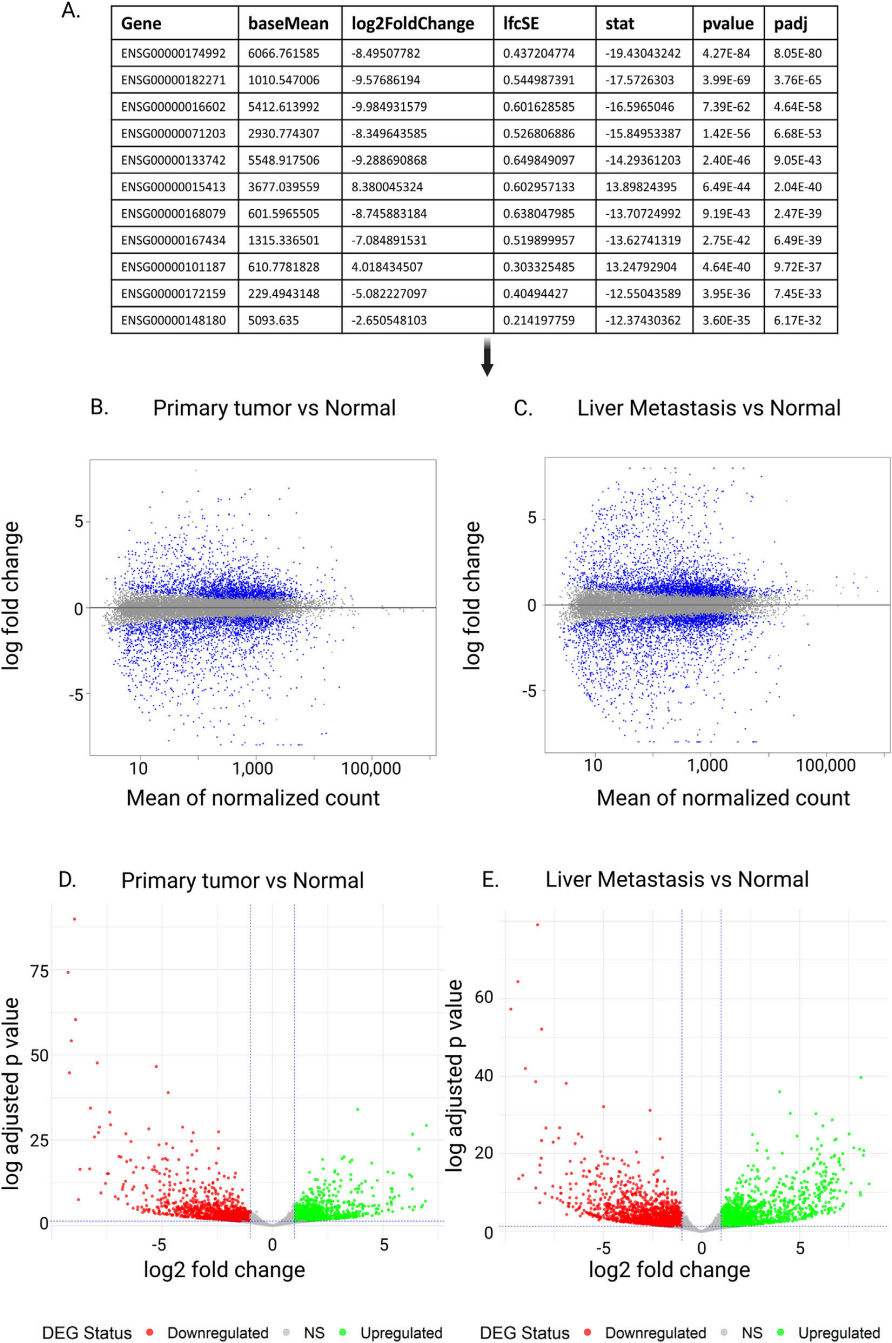
Exploration of inferences of differential expression analysis. **(A)** Output of DESeq2’s differential expression analysis. Each row represents a gene. In this case, Ensembl Gene ID is used. The baseMean column shows the average normalized expression level of the gene across all samples, which helps indicate if the gene tends to be highly or lowly expressed overall. The log2FoldChange value measures the expression difference between two conditions such as tumor versus normal tissue with a positive number signifying upregulation in the comparison group while a negative number indicates downregulation: for instance +1 means a twofold increase and −1 means a twofold decrease. The lfcSE provides the standard error of the log2FoldChange estimate indicating the reliability and precision of that measurement. The stat column displays the test statistic usually from a Wald test computed as the log2FoldChange divided by its standard error where higher absolute values point to stronger evidence of differential expression. The p-value reflects the likelihood that the observed expression change happened randomly assuming no actual difference exists. Given that many genes are analyzed at once, the adjusted p-value (padj or FDR) accounts for multiple comparisons to minimize false positives. A padj below 0.05 is generally viewed as statistically significant suggesting the gene is differentially expressed with strong confidence. **(B–E)** MA plot and volcano plot. **(B,C)** MA Plot comparing gene expression between primary tumor and normal **(B)** and liver metastasis and normal tissue samples **(C)**. The x-axis represents the mean of normalized counts, while the y-axis shows the log fold change. Blue and grey points indicate individual genes, with significantly differentially expressed genes and those do not, respectively. Volcano plots **(D,E)** illustrating differential gene expression between Primary Tumor and normal tissue **(D)**, and Liver metastasis and normal tissue samples **(D)**. The x-axis shows the log2 fold change, while the y-axis represents the −log10 adjusted P-value. Red dots indicate downregulated genes, green dots indicate upregulated genes, and gray dots represent non-significant (NS) genes.

The code below is part of the DESeq2 workflow. The first command, “dds < - DESeq (dds),” takes the dataset object created earlier (see above code) and tests whether expression differences between experimental groups are statistically significant. The next command, “res < - results (dds),” extracts the results table from the analysis (Figure 6A).

*dds < - DESeq (dds)*
*res < - results (dds)*

## 6 Exploration of inferences

A DESeq2’s output reports fold changes in gene expression between sample groups along with q-values for all genes tested (Figure 6A). We next demonstrate how these results can be visually explored to better interpret patterns of differentially expressed genes using a case study with three normal colon samples, three colorectal tumor samples, and three liver metastatic samples originally generated by Kim et al. (2014).

### 6.1 MA plots

Figures 6B,C present MA plots, which visualizes the relationship between the average expression level of each gene (A) and its log fold change (M). This type of plot is useful for spotting systematic biases, e.g., whether genes with very low or very high expression levels appear to be detected to change more frequently than others (Love et al., 2014; Robinson and Oshlack, 2010). In an expected plot, most genes cluster tightly around the horizontal zero line, reflecting no major change in expression between groups. If these densely clustered points form a curve or drift away from zero across the range of expression levels, it suggests a potential bias in the data that may require further correction.

The command below creates an MA plot from the results object (res) generated by DESeq2.

*plotMA (res)*

### 6.2 Volcano plots

While MA plots are useful to assess the dependency of gene expression levels on detected genes, volcano plots examine the relationship between statistical significance and estimated fold change of gene expression (McDermaid et al., 2019; Ritchie et al., 2015). Figures 6D,E show example volcano plots. In each plot, the horizontal dashed line marks the significance threshold (p-value = 0.05). Genes below this line are not statistically significant, and they typically also have small fold changes between groups. The vertical dashed lines mark the threshold for biologically meaningful fold changes—genes that fall close to the center (near zero on the x-axis) represent small expression differences, while those farther away represent larger changes. On the left side of the plot, negative fold-change values indicate downregulated genes (lower expression in tumor or metastasis compared to normal), whereas on the right side, positive values indicate upregulated genes (higher expression in tumor or metastasis). Genes that are neither significant nor strongly changing are shown in grey. Together, this creates a “volcano” shape: many genes cluster near the center with small changes, while fewer genes stand out at the top left or top right as highly significant and strongly differentiated. If the volcano plot does not show this general pattern, it may signal issues with the data or analysis that need further investigation.

The code below uses the ggplot2 package and creates a volcano plot. The second line sets up the plot using the results table (res) from DESeq2, where values for x- and y-axis are assigned through “aes (x = log2FoldChange, y = -log10 (padj)).”

*library (ggplot2) #load ggplot2*
*ggplot (res, aes (x = log2FoldChange, y = -log10 (padj)))*

### 6.3 Heatmap

While volcano plots and MA plots are useful for visualizing individual differentially expressed genes detected, they do not show overall expression patterns across samples. A heatmap complements these plots by displaying the expression levels of selected genes across all samples at once (Figures 5C,D). In a heatmap, genes and samples are clustered based on similarities in their expression patterns (McDermaid et al., 2019). This reveals groups of co-expressed genes and separates samples with distinct expression profiles, such as healthy and tumor tissues.

The accompanying dendrogram illustrates how items are grouped according to similarity of gene expression patterns, so that closely related samples or genes cluster together (Doyle, 2018). The grid colors represent scaled expression levels, with a smooth gradient ranging from low to high (Zhao et al., 2014). This allows researchers to see biological patterns and relationships that are missed when only looking at individual gene statistics (Figure 6).

The command, “pheatmap (VST_selected),” generates a heatmap using VST counts for a subset of genes of interest (VST_selected), which are selected from the full VST table described above.

*library (pheatmap) #load pheatmap*
*pheatmap (VST_selected)*

## 7 Experimental validation after data analysis

DGE analysis allows researchers to generate biological hypotheses about how genes behave under different conditions. For example, in a cancer study comparing tumor tissue with adjacent normal tissue, DGE might reveal that several oncogenes (e.g., MYC or KRAS) are upregulated, while tumor suppressor genes (e.g., TP53 or RB1) are downregulated. From this, one could infer that tumor cells have reprogrammed their transcriptional landscape to promote cancer initiation and progression. However, these findings are initial statistical inferences and do not confirm causation or even guarantee that the changes are biologically real.

Validation is, therefore, a critical next step. At the molecular level, RNA-Seq gives you a global snapshot of gene expression, but technical noise, sequencing depth, alignment biases, and statistical modeling can all influence the results (Alwine et al., 1977). Therefore, validation is necessary for detected genes using a more precise method. For example, quantitative PCR (qPCR) is suitable for validation because it amplifies and quantifies the expression of each gene (Bustin et al., 2009). Similarly, Northern blotting can be used, which measures RNA quantity and assesses its size and integrity by separating samples via gel electrophoresis, transferring them to a membrane, and probing with labeled sequences. Also, Western blotting and immunohistochemistry are appropriate if protein-level changes are relevant. Alternatively, results can also be tested in additional biological replicates or independent datasets to rule out sample-specific effects.

For functional validation, one might manipulate the expression of candidate genes to directly test their role in disease. For instance, if RNA-Seq analysis suggests that the oncogene MYC is upregulated in tumors compared to normal tissue, functional experiments could involve silencing MYC using siRNA or CRISPR-based approaches. If reduced MYC expression slows cell proliferation in tumor cells, this would support the inference that MYC overexpression contributes to tumor growth.

Together, these steps transform RNA-Seq results from statistical observations into validated biological knowledge. In this sense, DGE analysis acts as a powerful discovery tool that points researchers toward promising targets, but rigorous validation ensures that the conclusions are robust and reproducible.

## 8 Conclusion

RNA-Seq has become an indispensable tool for exploring the transcriptome, allowing researchers to scan the entire landscape of gene expression and generate new biological hypotheses. As the integration of RNA-Seq into molecular biology research continues to expand, it is increasingly important for molecular biologists to understand the foundations of RNA-Seq analysis. This includes not only the basic workflow but also the statistical principles that ensure scientific rigor. This review aims to serve as a fundamental guide for beginners.

In addition to synthesizing current knowledge, this review paper can be used as a teaching resource for advanced undergraduate and graduate-level education in genomics, bioinformatics, and molecular biology. To facilitate active learning, we developed a lesson plan that integrates this review as pre-class reading, a quiz to assess comprehension and reinforce key concepts, and a hands-on R scripting lab for applying DGE analysis in practice. These materials (lesson plan, example quiz questions with answers, and R scripts) are made available for others to use (Supplementary Material and https://github.com/dprabin25/BegineersRNA-Seq).
