# Supplementary material for: From bench to bytes: a practical guide to RNA sequencing data analysis
Source: Front Genet. 2025 Oct 28;16:1697922. doi: 10.3389/fgene.2025.1697922 (PMC12599993; doi:10.3389/fgene.2025.1697922)
Supplement: Supplementary file 1 [file DataSheet1.docx]

**Supplementary Data**

**Lesson Plan: Differential Expression Analysis with RNA-Seq**

***Learning Objectives***

By the end of this session, students will be able to:

- Explain the workflow of RNA-seq data analysis.
- Interpret key visualization outputs.
- Apply DESeq2 in R to perform basic differential expression analysis.
- Critically evaluate sources of bias and limitations in RNA-seq analysis.

***Pre-Class (Homework)***

Assigned reading: The main text (this review paper).

Deliverables: Students are expected to take notes on key steps.

***In-Class (90 minutes total)***

1. Quiz & Discussion (15 min)

Format: 5–7 multiple-choice or short-answer questions based on the review paper. Example quiz questions are available in this document (see below)

Purpose:

- - Ensure students understand the key workflow steps.
  - Surface common misconceptions.

Activity:

- - Students answer individually.
  - Students have a short peer discussion.
  - Instructor reviews answers with an explanation.

1. Transition Mini-Lecture (10 min)

- Brief recap of key concepts revealed by the quiz.
- Emphasis on why normalization and multiple-testing correction are critical.
- Link conceptual review → coding application.

1. Hands-On R Lab (50 min)

Setup: Students open the provided R script (from <https://github.com/dprabin25/BegineersRNA-Seq>).

Activities:

- Load example count data (Kim et al., 2014 dataset).
- Explore the count data format.
- Run DESeq2 normalization.
- Perform DE analysis between normal, tumor, and metastasis.
- Generate and interpret volcano plots, MA plots, heatmap, and PCA.

Instructor role: Walk around, troubleshoot, encourage peer support.

1. Wrap-Up & Reflection (15 min)

Quiz:

Format: 5–7 multiple-choice or short-answer questions based on the hands-on R lab. Example quiz questions are available in this document (see below)

Group discussion:

- What challenges did you encounter?
- How do these methods help us avoid false discoveries?
- How might you extend this pipeline (e.g., pathway analysis)?
- Exit ticket: Each student writes one “aha” moment + one remaining question.

***Assessment***

Pre- and post-quiz, exit ticket, and in-class lab participation.

**Example quiz questions**

**A. 1. Raw count data**


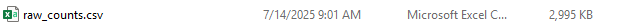


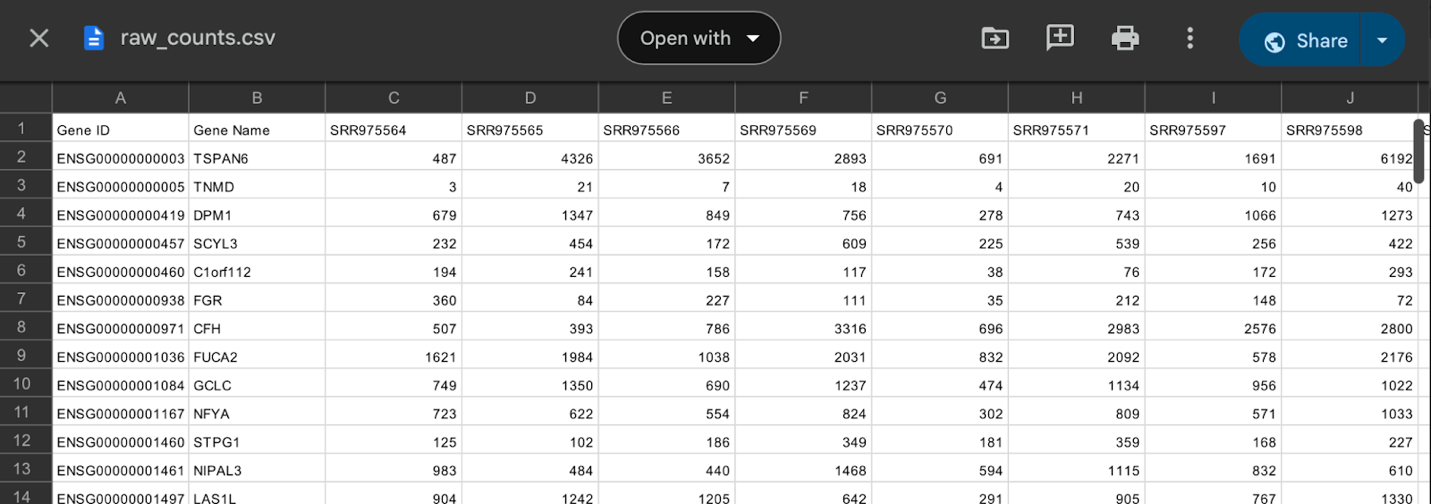


**A. 1. (i) Is raw count data a CSV, TSV, or Excel file?**

**Answer:** Common formats for raw count data include CSV (.csv), TSV (.tsv or .txt), and occasionally Excel (.xlsx) files. CSV files use commas to separate values, while TSV files use tabs; among these, TSV is the most used format in bioinformatics due to its compatibility with command-line tools and scripting languages like R and Python. Although Excel files are sometimes used for data sharing or visualization, they are generally not recommended for reproducibility or automated workflows, as they can introduce formatting issues and are less compatible with many bioinformatics pipelines.

**A. 1. (ii): Is raw count gene-by-sample or sample-by-gene? (What are the practices?)**

**Answer:** The standard practice is Gene-by-sample, that means: Rows = Genes, Columns = Samples.

**A. 1. (iii): Does the file include header rows/column names?**

**Answer:** Yes, the file should include: A header row with sample names, a first column with gene IDs, and a second column with Gene names.

**A. 2: Contents in raw count**

**A. 2. (i): What does each row represent? (e.g., gene, transcript, feature)**

**Answer:** Each row commonly represents a gene, but it can also be a transcript, exon, or other genomic element, depending on the quantification tool.

**A. 2. (ii): What does each column represent? (e.g., sample names)**

**Answer:** Each column represents a sample, typically labeled with unique sample names (e.g., Control_1, Treated_2).

**A. 2. (iii): Are the values raw counts or normalized counts? How do you differentiate?**

**Answer**: Typically, values in the raw count data file are *raw counts*—the number of sequencing reads mapped to each gene or feature in each sample.

Differentiation criteria

| **Criteria** | **Raw Counts** | **Normalized Counts** |
| --- | --- | --- |
| **Value Type** | Whole numbers (e.g., 0, 45, 212) | Decimals or scaled numbers (e.g., 5.23) |
| **Generated by** | Tools like HTSeq and featureCounts | Tools like DESeq2, edgeR, or using TPM/RPKM/FPKM |
| **Usage** | Input for differential expression analysis | Input for differential expression analysis when a tool requires normalized counts |
| **File Labeling (optional)** | Often named raw_counts.csv, counts.txt | May include TPM, normalized, and RPKM in the filename |
|  | 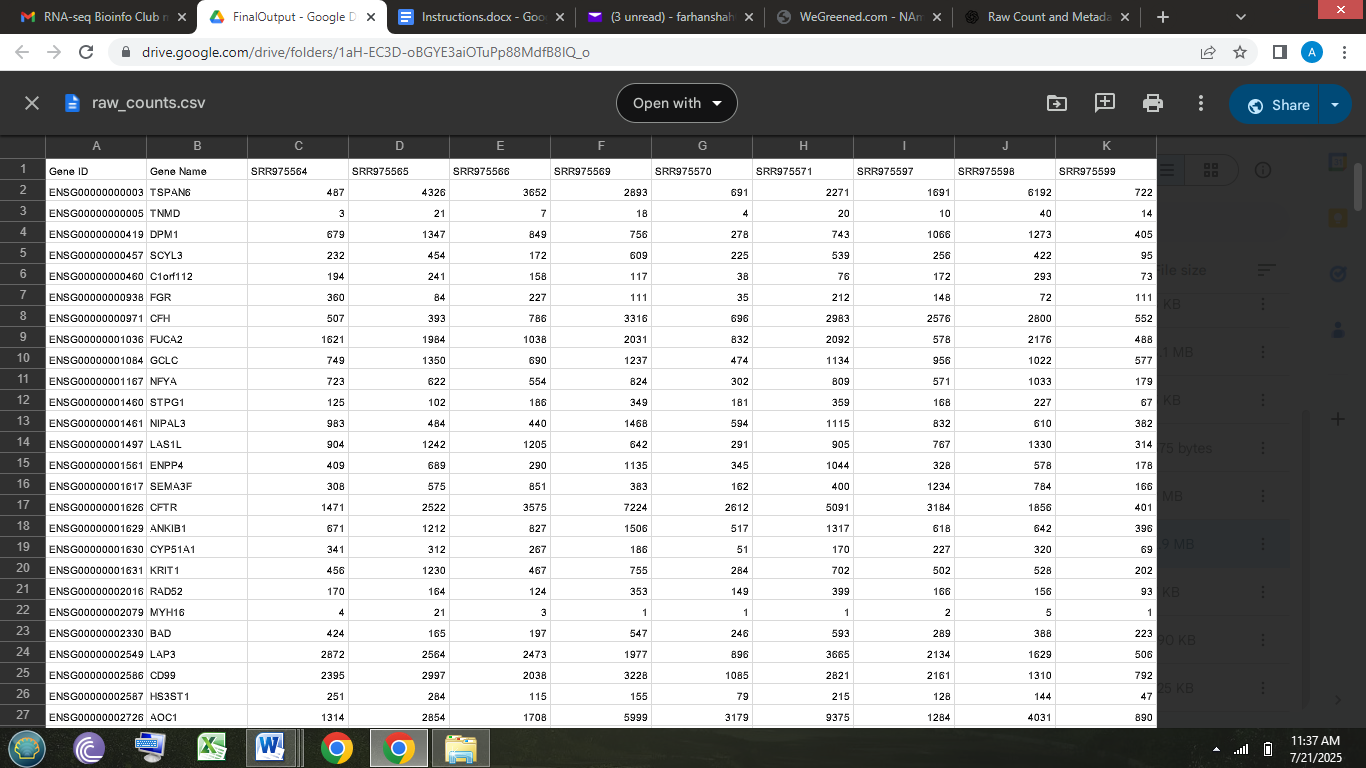 | 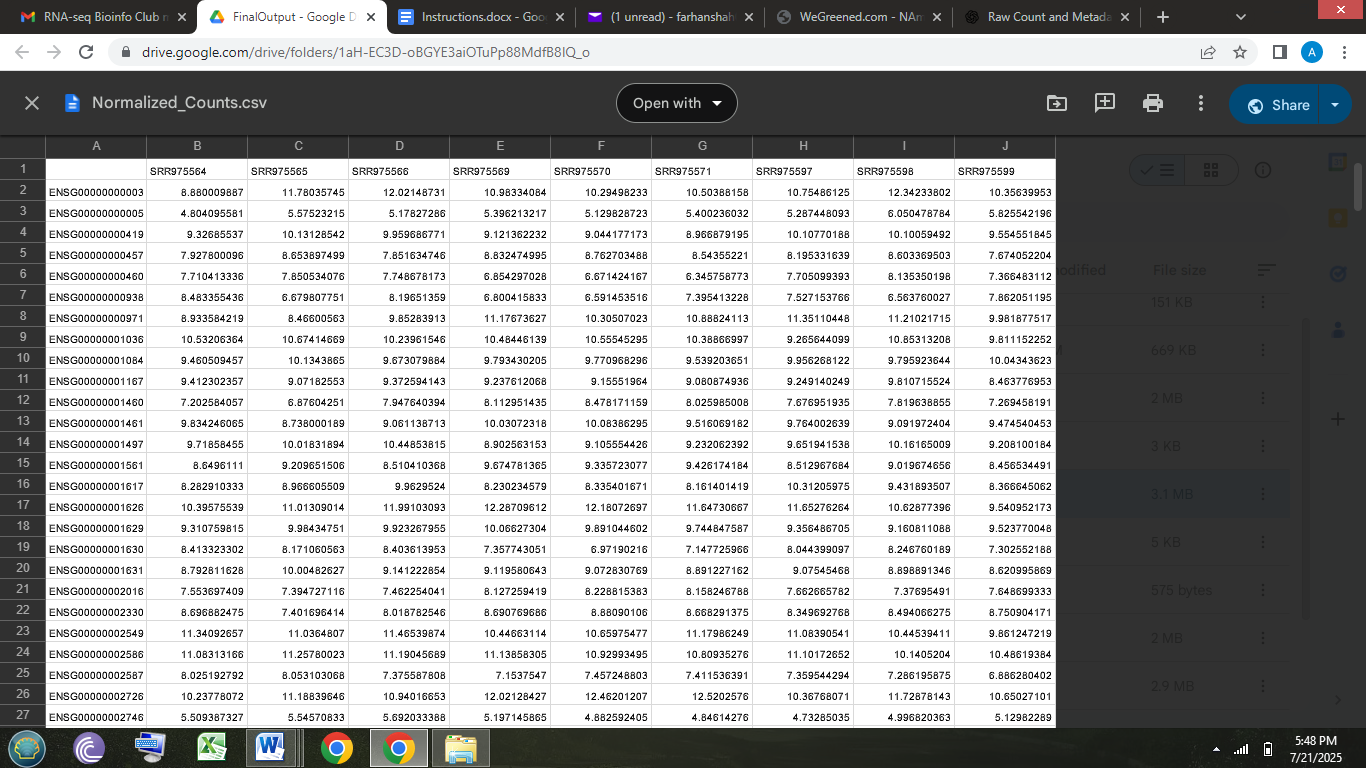 |

**A. 3: Gene identifiers**

**A. 3. (i): Are the gene identifiers Ensembl IDs, gene symbols, or something else?**

**Answer:** Gene identifiers in raw count data can be Ensembl IDs, gene symbols, or other types, depending on the annotation source used during read quantification.

Ensembl IDs (e.g., ENSG00000139618) are unique, stable, and widely used in RNA-seq pipelines.

Gene symbols (e.g., BRCA2, ACTB) are more human-readable but can be ambiguous due to synonyms or updates.

Other identifiers, like Entrez IDs or custom feature IDs, may appear in specialized or non-model organism datasets.

Best practice is to use Ensembl IDs for raw analysis and convert to gene symbols later for interpretation and visualization.

**A. 4: Missing values or NA entries**

**A. 4. (i): Are any count values missing or NA?**

**Answer:** Missing values (NA) are generally not present in raw count matrices. If you see **NA**, it may indicate an issue during read counting or data export.

**A. 4. (ii): How are zeros represented (true zero or missing data)?**

**Answer:** Zeros represent true biological or technical zeros, which means no reads were mapped to that gene in that sample. Zeros are not considered missing, as they are meaningful and should be retained for downstream analysis.

**A.5: Samples and genes**

**A. 5. (i): How many samples and genes are there?**

**Answer:** The number of samples equals the number of columns (excluding the first column for gene IDs), and the number of genes equals the number of rows.

**A. 6: Sample names**

**A. 6. (i): Do sample names reflect conditions, replicates, or time points?**

**Answer:** Yes, Sample names often reflect experimental conditions, replicates, or time points.

**A. 6. (ii): Is the metadata in a separate file or encoded in the sample names? (Also can be prepared while writing the analysis code, or metadata can be a separate file)**

**Answer:** Metadata uses a separate metadata file (CSV/TSV) that includes sample names, conditions, time points, batches, etc., which can be linked during data analysis.

**A. 7: Gene filter**

**A. 7. (i): Are low-count genes already removed?**

**Answer**: Not usually. Raw count data typically includes all genes, including those with very low or zero counts across most samples. Filtering is often done during the preprocessing stage before differential expression analysis.

**A. 7. (ii): How does filtering affect the accuracy and power of RNA-seq statistical tests (like DESeq2 or edgeR)?**

**Answer:** Filtering out low-count genes improves:

- Statistical power by reducing the multiple testing burden (fewer false positives)
- Variance estimation by removing noisy, low-information features
- Model fit by focusing on genes with meaningful expression patterns

Both DESeq2 and edgeR recommend pre-filtering genes with very low total counts.

**A. 7. (iii): What are the risks of setting the filtering threshold too high or too low?**

**Answer:** Too low threshold (keeping too many low-count genes):

- Retains noisy, uninformative genes
- Increases false positives due to high variability and low signal
- Reduces statistical power by increasing the number of tests (multiple testing burden)
- May distort normalization and dispersion estimates

Too high threshold (removing too many genes):

- Risk of discarding biologically important genes, especially:
  - Lowly expressed transcription factors, non-coding RNAs, or rare transcripts
- Can reduce the discovery of condition-specific or tissue-specific signals
- May bias results toward abundant housekeeping genes

Use a moderate threshold, like keeping genes with a minimum count (e.g., ≥10) in at least 2 or more samples. This balances noise reduction with retaining biologically relevant genes.

**A. 7. (iv): How does filtering affect the accuracy and power of RNA-seq statistical tests (like DESeq2 or edgeR)?**

**Answer:** Filtering low-count genes improves both accuracy and statistical power in RNA-seq analysis:

Positive effects of filtering are

**Reduces noise**

- Low-count genes often show high variability and poor reproducibility.
- Removing them prevents random variation from skewing results.

**Improves dispersion estimation**

- Tools like DESeq2 and edgeR estimate gene-wise dispersion. Including many low-count genes can distort these estimates.

**Increases statistical power**

- Fewer genes = fewer tests = less multiple testing correction → more chance to detect truly differentially expressed (DE) genes.

**Enhances normalization accuracy**

- Filtering removes extreme or unstable values that can bias size factor or scaling calculations.

**If filtering is skipped or poorly done:**

- Increases false discovery rate (FDR)
- Weakens the ability to detect true DE genes
- Slows down computation unnecessarily
- May cause unstable p-values and fold-change estimates

Filtering before analysis helps DESeq2 and edgeR focus on genes with reliable, informative expression, boosting accuracy, interpretability, and biological relevance of results. (Niedziela, G.,2022).

**A. 7. (v): Are there any biological reasons to keep certain low-count genes (e.g., transcription factors, rare transcripts)?**

**Answer:** Yes, absolutely. Low-count genes can be biologically important, and removing them without consideration may result in the loss of key insights.

Biological reasons to keep low-count genes:

1. Transcription factors (TFs):
   - Often expressed at low levels, but regulate hundreds of downstream genes.
   - Small expression changes can have large regulatory effects.
2. Signaling molecules and receptors:
   - Genes like cytokines or membrane-bound receptors can be low in abundance but critical in pathways (e.g., immune response).
3. Rare or cell-type–specific transcripts:
   - In bulk RNA-seq, transcripts from rare cell populations can appear low-count but still reflect meaningful biological processes.
4. Non-coding RNAs:
   - miRNAs, lncRNAs, and snoRNAs are typically expressed at lower levels but are involved in gene regulation, splicing, and translation.
5. Early response or condition-specific genes:
   - Some genes are transiently or weakly expressed only under certain conditions (e.g., stress, infection).

So, recommendations are

- To use adaptive or flexible filtering: For example, filter by count and require expression in at least a few samples rather than a hard cutoff across all.
- If you expect key regulatory genes, consider keeping a curated list of genes to retain, even if they fall below the threshold.

**A. 7. (vi): Does the choice of gene filter threshold affect downstream results like clustering or PCA?**

**Answer:** Yes, it has a significant impact.

How gene filtering affects clustering and PCA:

1. Too lenient (low threshold):
   - Retains many low-count, noisy genes
   - Introduces random variation and technical noise
   - Obscures true biological patterns in PCA or clustering
   - May lead to poor sample separation and misleading groupings
2. Too strict (high threshold):
   - Removes informative low-expression genes (e.g., regulatory factors)
   - Reduces biological signal and diversity of expression profiles
   - Can cause samples to cluster too tightly, masking subtle differences
3. Balanced filtering:
   - Enhances signal-to-noise ratio
   - Improves the separation of biologically meaningful groups in PCA and clustering
   - Allows detection of both major and subtle trends

Best practice:

- Filter genes with minimal expression across most samples (e.g., keep genes with a count ≥10 in at least 2 or 3 samples).
- If clustering or PCA is a major goal, consider applying variance-based filters (e.g., keep the top 500 most variable genes).

Summary:

The gene filtering threshold directly influences the clarity, reliability, and biological interpretability of downstream exploratory analyses like PCA and clustering. Choose a threshold that balances noise reduction with information retention.

**A. 7. (vii): Should the filtering be done before or after normalization?**

**Answer:** Filtering should be done *before* normalization.

Why filter before normalization?

1. Improves normalization accuracy
   - Low-count genes can distort size factor or scaling estimates used during normalization (especially in DESeq2 or edgeR).
   - Removing uninformative genes first ensures more stable and representative normalization factors.
2. Reduces noise early
   - Prevents noisy, low-expression genes from influencing downstream steps (e.g., dispersion estimation, variance calculations).
3. Efficient processing
   - Reduces computational load by excluding genes unlikely to be biologically meaningful or statistically significant.

If you filter after normalization:

- The normalization process may be biased by uninformative or zero-count genes, affecting downstream results like differential expression, clustering, and PCA.

Best practice:

- Filter genes with very low counts in most or all samples (e.g., keep genes with counts ≥10 in at least 2 or 3 samples).
- Then, apply normalization using the filtered dataset (McCarthy, D. J., 2017).

**B. Metadata structure**


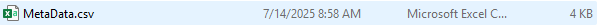


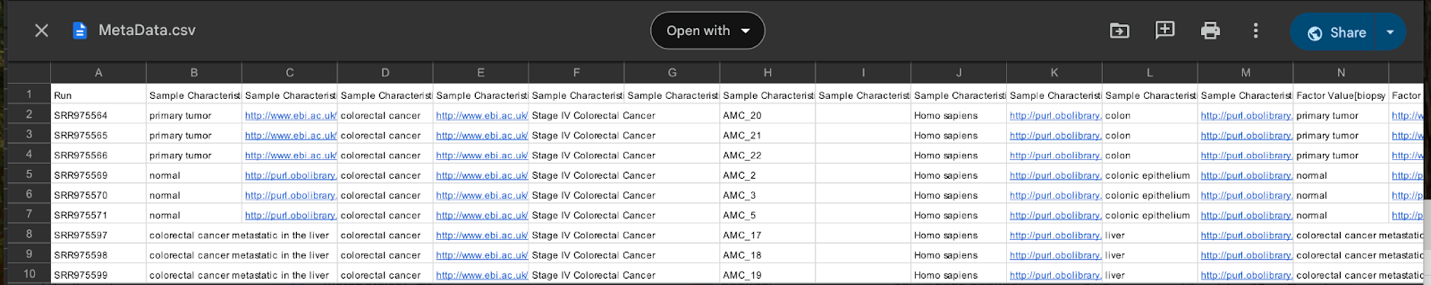


**B. (i) What is metadata? Why is it important?**

**Answer:** Metadata in RNA-seq refers to the accompanying information that describes each sample in the experiment, such as condition, treatment group, time point, tissue type, or batch. It is crucial because it defines the experimental design and enables proper grouping and statistical comparisons in tools like DESeq2 or edgeR.

**B. (ii): Do the sample names in the metadata file exactly match the column names (excluding the gene column) in the raw count matrix?**

**Answer:** Yes, this is essential. If the names don’t match exactly, the software (e.g., DESeq2, edgeR) won’t be able to correctly associate metadata with the count data, which will cause errors or mismatched analyses.

**B. (iii): Are all samples in the count matrix present in the metadata, and vice versa?**

**Answer:** Yes, every sample appearing in your count matrix should have a corresponding entry in the metadata, and no “extra” samples should be present in either file. Missing samples will result in dropped data or alignment errors.

**B. (iv): Are the sample names written in the same format (case-sensitive, no extra spaces)?**

**Answer:** Yes, software treats *Sample1* and *sample1* as different entities. Even trailing spaces or hidden characters can break matching. Always keep formatting consistent (case, underscores, hyphens).

**B. (v): Do both files have the same number of samples (columns in count data = rows in metadata)?**

**Answer:** Yes, the number of samples in your count matrix (excluding the gene column) should match the number of rows in your metadata exactly. Mismatches indicate missing or extra samples and must be fixed before running the analysis.

**B. (vi): Are biological replicates properly indicated in the metadata (e.g., Rep1, Rep2, Rep3)? (Note: But our samples were not replicates.)**

**Answer:** Biological replicates are typically indicated in metadata (e.g., Rep1, Rep2), if there are no replicates—as in your case—this can be noted clearly, but other experimental variables must still be properly defined. Structuring metadata accurately is critical because any mismatch can lead to errors in analysis or misinterpretation of results.

**C. Normalized count**


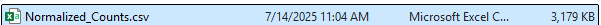


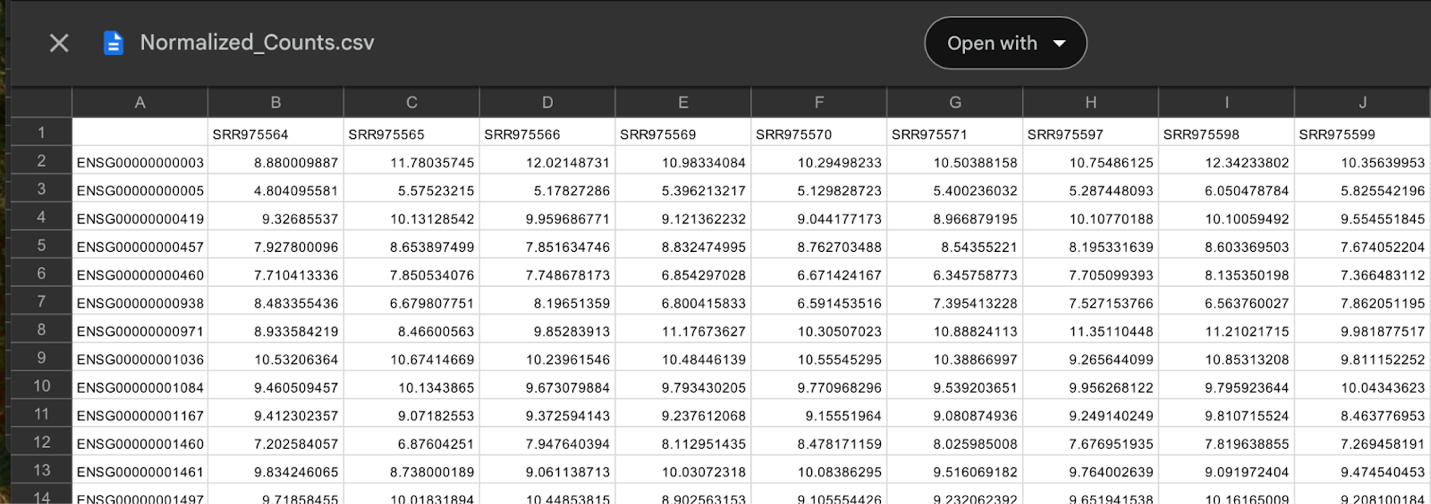


**C. (i): What does a normalized count matrix typically look like?**

**Answer:** A normalized count matrix is a CSV or TSV file where:

- Rows = genes (or transcripts)
- Columns = samples
- Values = normalized expression values (usually non-integer and comparable across samples)

**C. (ii): What are the differences between raw counts and normalized counts?**

**Answer:** Raw counts are the unprocessed, integer values representing the number of sequencing reads mapped to each gene in each sample. They reflect both biological expression levels and technical factors like sequencing depth.

Normalized counts are adjusted values that account for differences in sequencing depth (and sometimes gene length), allowing for fair comparison across samples. These values are typically non-integer and scaled to make gene expression levels more comparable between samples.

**C. (iii): How does normalization account for sequencing depth and gene length?**

**Answer:**

**Sequencing depth:**

Normalization adjusts for differences in how many total reads were generated for each sample. For example, DESeq2 calculates *size factors* to scale counts so samples with more reads aren’t artificially inflated. Similarly, edgeR uses *TMM normalization* to correct for library size and composition bias. This ensures counts are comparable across samples regardless of how deeply each was sequenced.

**Gene length:**

Methods like TPM (Transcripts Per Million) and RPKM/FPKM also adjust for gene length, because longer genes naturally accumulate more reads. They divide the read counts by gene length in kilobases so expression levels reflect transcript abundance rather than gene size.

**C. (iv): How do the distributions of normalized counts compare across samples?**

**Answer:** After normalization, the distributions of gene expression counts across samples should be more similar and comparable. Normalization reduces technical variation (like differences in sequencing depth) so that biological differences stand out more clearly.

Before normalization, samples with higher sequencing depth may show inflated count values, leading to wider or skewed distributions.

**C. (v): When should you use scaled counts (e.g., for PCA, heatmaps, clustering)?**

**Answer:** We should use scaled counts when performing exploratory and visualization-based analyses, such as:

- PCA (Principal Component Analysis)—to reduce dimensionality and compare global expression patterns across samples
- Heatmaps—to visualize gene expression levels and clustering of genes/samples
- Hierarchical clustering or k-means—to group samples based on expression profiles
- Correlation analysis—to assess similarity between samples or genes

**C. (vi): Should you use normalized or raw counts for differential expression analysis?**

**Answer:** We should use raw counts as the input for differential expression analysis with tools like DESeq2 or edgeR.

These tools require raw, integer counts because they perform their own internal normalization and statistical modeling based on assumptions about count distributions (e.g., negative binomial). Supplying pre-normalized or non-integer data can lead to incorrect results.

**C. (vii): What do large or small normalized counts indicate about gene expression levels?**

**Answer:** Large normalized counts indicate high gene expression, meaning the gene is abundantly transcribed in that sample. These are typically housekeeping genes or genes that are strongly active under certain conditions.

Small normalized counts indicate low gene expression, suggesting the gene is weakly transcribed, inactive, or only expressed in specific cell types or conditions.

Consistently high counts across all samples may suggest a constitutively expressed gene, while variable counts between groups could indicate a differentially expressed gene.

**D. DEG analysis**


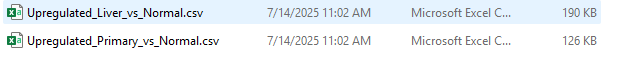


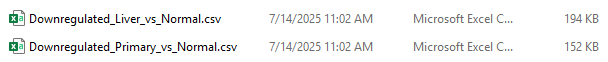


**
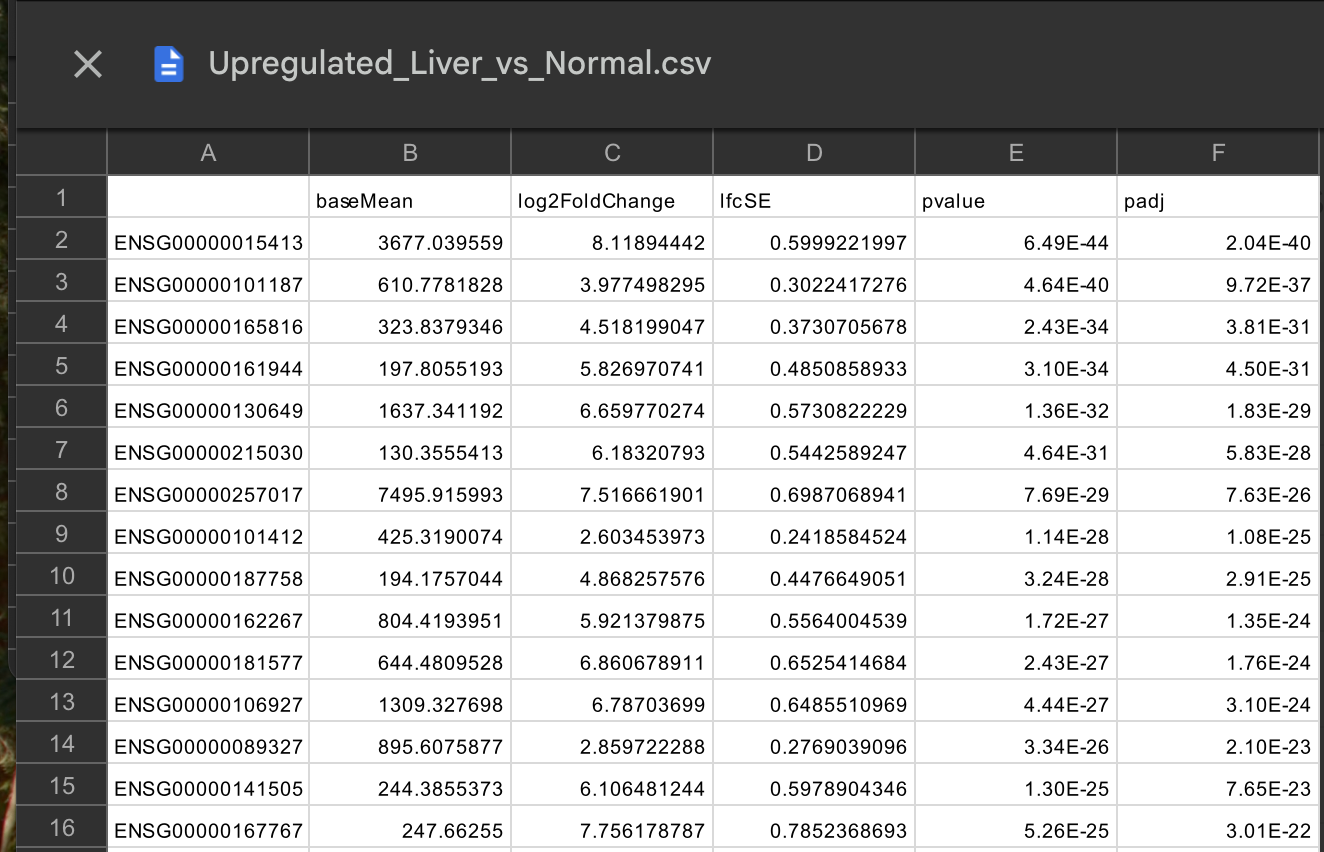
**

**D. (i): What is differential expression (DE) analysis?**

**Answer:** DE analysis identifies genes that show statistically significant changes in expression between two or more biological conditions (e.g., control vs. treated).

**D. (ii): What is the goal of DEG analysis in RNA-seq?**

**Answer:** To determine which genes are upregulated or downregulated between conditions, providing insight into biological processes or pathways that respond to treatments, diseases, or time points.

**D. (iii): What does it mean for a gene to be differentially expressed?**

**Answer: A** gene is differentially expressed if its expression level significantly changes between groups—either increased (upregulated) or decreased (downregulated)—beyond what would be expected by chance.

**D. (iv): What is the role of false discovery rate (FDR) or adjusted p-values?**

**Answer:** RNA-seq tests thousands of genes, increasing the chance of false positives.

FDR, or adjusted p-value (padj), corrects for multiple testing (e.g., using Benjamini-Hochberg correction).

It controls the proportion of false discoveries among genes called significant.

**D. (v): What does the resulting CSV file contain? (e.g., log2FC, p-value, padj)**

**Answer:** The DEG result file typically includes

| **Column** | **Meaning** |
| --- | --- |
| log2FoldChange (log2FC) | Magnitude & direction of expression change |
| p-value | Raw statistical significance |
| padj (adjusted p-value) | Corrected significance after multiple testing |
| baseMean | Average expression across all samples |
| stat | Test statistic used in the model |

**log2FC** tells you if a gene is upregulated or downregulated and by how much.

**padj** tells you how statistically reliable the observed difference is.

**baseMean** helps assess expression level context (e.g., high vs. low expressed genes).

**D. (vi): What is the size factor? Importance of the size factor?**

**Answer:** The Size factor is used in tools like DESeq2 to normalize RNA-seq data. It accounts for differences in sequencing depth or library size between samples.

**Importance of the size factor:**

- RNA-seq generates different total counts per sample (some have more reads).
- Without correction, samples with more reads appear to have higher expression.
- The size factor scales raw counts so that expression is comparable across samples.
- DESeq2 automatically estimates size factors during preprocessing.

**D. (vii): How should you interpret the log2 fold change (LFC)?**

**Answer:**

log2FC = 1 → 2-fold upregulation

log2FC = -1 → 2-fold downregulation

Values near 0 = no meaningful change

The sign indicates direction (positive = upregulated in group 2)

**D. (viii): What are the typical thresholds for calling genes significant?**

**Answer:**

padj < 0.05 → statistically significant

|log2FC| > 1 → biologically meaningful change (2-fold up/down)

Some analyses use stricter or more relaxed thresholds depending on the goal.

**D. (ix): What are the biological groups being compared (group1, group2)?**

**Answer:** These are the experimental conditions, like

- Treated vs. Control
- Infected vs. Healthy
- Time point 1 vs. Time point 2

Defined during metadata setup and used in the DE model.

**D. (x): Why might one group show more DEGs than another?**

**Answer:** The treatment had a stronger biological effect in one group.

There may be higher variability in one group.

Sample size differences or quality issues could affect statistical power.

**D. (xi): What are the biological implications of upregulated vs. downregulated genes?**

**Answer:** Upregulated genes → More active under a condition; may play key roles in that condition (e.g., immune response).

Downregulated genes → Less active; might reflect suppressed pathways or loss of function.

These help identify affected pathways, cellular processes, or biomarkers.

**E. PCA**


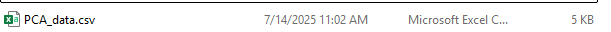


**E. (i) What are PC1 and PC2? How are they calculated?**

**Answer:** PC1 (Principal Component 1) and PC2 (Principal Component 2) are the first two axes in a Principal Component Analysis (PCA).

They capture the major sources of variation in gene expression data:

- PC1 = direction of the greatest variance (most significant difference).
- PC2 = the next highest variance, orthogonal to PC1. For its calculation, start with normalized gene expression data, then center the data (subtract the mean from each gene). After that, calculate the covariance matrix (how genes co-vary). Use eigenvalue decomposition or SVD to extract:
- Eigenvectors → principal components (PC1, PC2, etc.).
- Eigenvalues → amount of variance each component explains.

Samples are projected onto PC1 and PC2 to visualize patterns (like clustering by condition).

**E. (ii): What do those values represent?**

**Answer:** Each sample gets a coordinate (score) on PC1 and PC2. These scores represent how much that sample aligns with the underlying expression patterns captured by PC1 and PC2. If samples group on a PCA plot, it means their overall gene expression profiles are similar.

**E. (iii): What do positive and negative values represent?**

**Answer:** Positive and negative values reflect the direction of variation. For example, samples with positive PC1 values might be "treated," while negative PC1 values could be "control." The magnitude shows how strongly a sample expresses the gene pattern associated with that PC. Interpretation depends on context—the biological or technical variable that explains the separation.

**E.(iv): What should a PCA plot look like (appearance)?**

**Answer:** A 2D scatter of samples with PC1/PC2 axes labeled with % variance and a clear legend.

**E. (v): What are PC1 and PC2 values in PCA?**

**Answer:** The fraction of total variability summarized by a component (e.g., PC1 = 32%).

**E. (vi): What are the points in PCA?**

**Answer:** Points = samples; coordinates are their PC1/PC2 scores (linear combinations of gene expression). Axes are the principal components.

**E. (vii): Why is PCA important in RNA-seq analysis?**

**Answer:** Rapid QC to spot outliers/batch effects and to verify expected group separation.

**E. (viii): How should biological groups behave on a PCA plot?**

**Answer:** Replicates clusters; distinct groups separate along one or more PCs if biology dominates noise.

**F. Volcano plot**

*Example of a volcano plot*


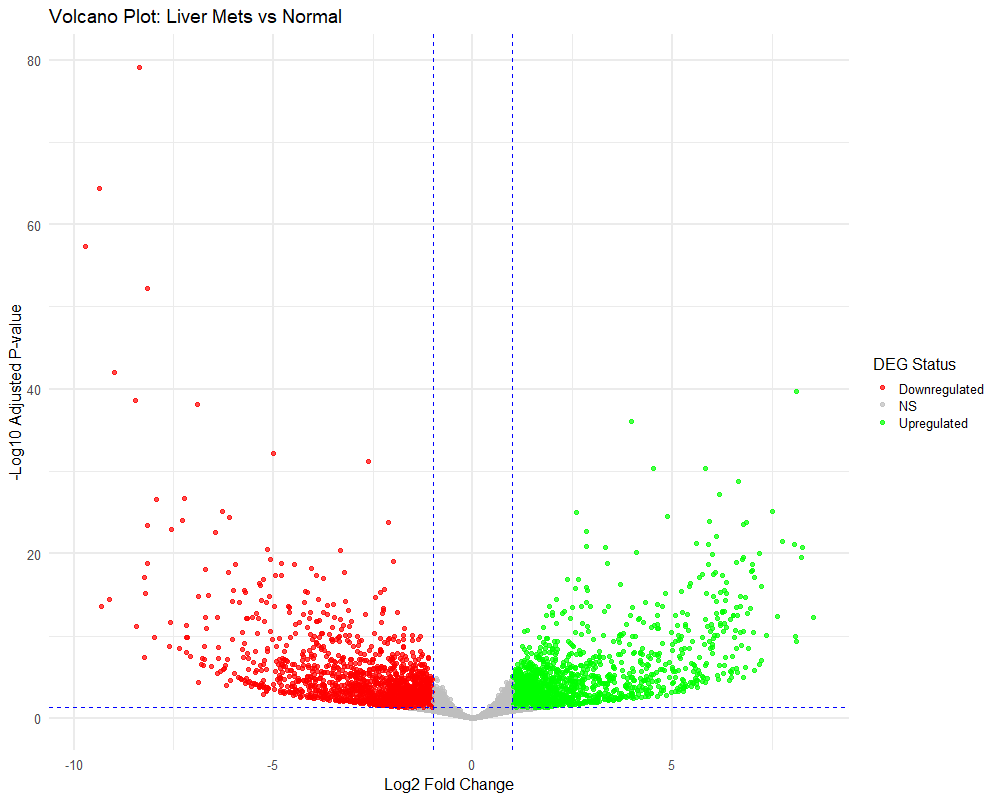


**F. (i): What are shown in a volcano plot?**

**Answer:**

A volcano plot is a type of scatter plot used in differential gene expression analysis to visualize both the magnitude of change and the statistical significance of each gene between two biological conditions. (Wentian et al., 2014).

Each dot represents a gene, and the position of the dot is determined by two values: the log2 fold change (LFC) on the x-axis and the –log10 adjusted p-value (usually FDR or padj) on the y-axis.

**F. (ii): Why is the plot shaped like a volcano?**

**Answer:** The plot is shaped like a volcano because most genes have little or no change in expression (clustered near the center of the x-axis), and only a subset of genes show large fold changes with high statistical significance, rising to the top left and top right like volcanic "arms."

**F. (iii): What does "left vs. right" mean? What does "higher vs. lower" mean on the plot? Which dots are significant?**

**Answer:** Genes on the left side have negative LFCs (downregulated in the test group), while those on the right have positive LFCs (upregulated in the test group). The higher a dot is on the y-axis, the more statistically significant the change (i.e., lower p-value).

**F. (iv): Why are some dots colored differently?**

**Answer:** Colored dots usually indicate statistically significant genes, based on thresholds such as |log2FC| > 1 and adjusted p-value < 0.05.

**F. (v): What are those horizontal and vertical lines for?**

The vertical lines represent the fold change cutoffs (e.g., log2FC = ±1), and the horizontal lines show the significance threshold (e.g., padj = 0.05).

**F. (vi): Is a gene with a small fold change still important if it's high on the plot?**

**Answer:** A gene with a small fold change but a very high position on the plot (very low p-value) can still be biologically meaningful, especially if it's involved in key pathways or regulatory processes. Likewise, large fold changes without statistical support (low on the plot) should be treated with caution. Overall, the volcano plot helps quickly identify which genes are most strongly and confidently differentially expressed.

**G. MA plot**

*Example of a MA plot*


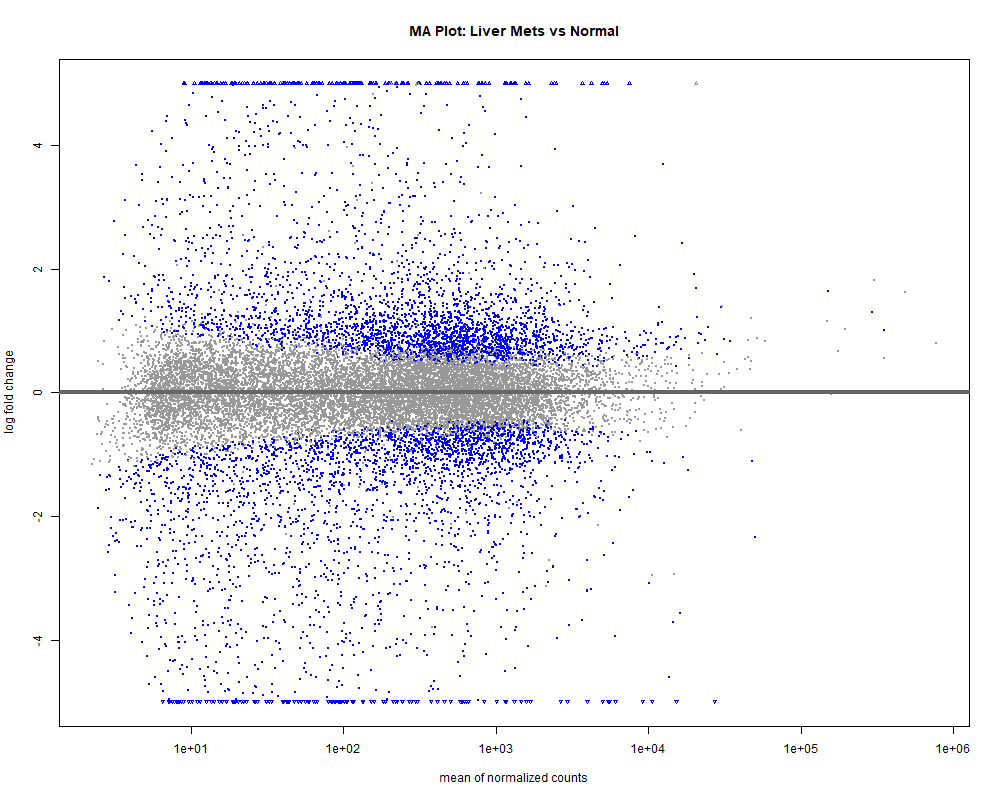


**G. (i): What does MA plot show?**

**Answer:** An MA plot is a graphical representation used in RNA-seq analysis to visualize the relationship between the magnitude of gene expression and the change in expression between two conditions.

**G. (ii): What do the X and Y axes represent?**

**Answer:** On the X-axis, it shows the average expression (A) of each gene across all samples, typically on a log scale. The Y-axis represents the log2 fold change (M), showing how much a gene’s expression changes between conditions.

**G. (iii): What are the points in a plot?**

**Answer:** Each dot in the MA plot represents a gene.

**G. (iv):** **Why is most of the data centered around zero?**

**Answer:** Most dots cluster around zero on the Y-axis because many genes are not significantly differentially expressed, meaning their expression doesn't change much between the two conditions.

**G. (v): What does a dot above or below the center mean?**

**Answer:** A dot above the center line indicates a gene that is upregulated, while a dot below means it is downregulated in the condition being tested.

**G. (vi): What does “average expression” mean on the X-axis?**

**Answer:** The average expression on the X-axis reflects how highly expressed the gene is across the samples.

**G. (vii): Why are some points colored differently?**

**Answer:** Some points are colored differently (usually red, blue, or other shades) to highlight genes that are statistically significant based on criteria like adjusted p-values and fold change thresholds.

**G. (viii): Are extreme dots more important?**

**Answer:** Extreme dots (far above or below the center) often indicate stronger differential expression and are more biologically interesting, though interpretation depends on context.

**G. (ix): How is this different from a volcano plot?**

**Answer:** Compared to a volcano plot, which emphasizes significance (p-value) versus fold change, the MA plot focuses on fold change vs. expression level. Volcano plots use the Y-axis for statistical significance, while MA plots use it for fold change, making MA plots more suited for examining the reliability of changes across expression levels.

**G.(x): What do the triangles above and below the center mean?**

**Answer:** Triangles on the top or bottom edges usually represent genes with fold changes too extreme to be shown fully on the plot—they are clipped to keep the scale readable but still marked to show their existence.

**References**:

Kim, S.K., Kim, S.Y., Kim, J.H., Roh, S.A., Cho, D.H., Kim, Y.S., and Kim, J.C. (2014). A nineteen gene-based risk score classifier predictsthe prognosis of colorectal cancer patients. *Molecular oncology, 8*(8):1653-1666. Doi: <https://doi.org/10.1016/j.molonc.2014.06.016>

Li, W., Freudenberg, J., Suh, Y. J., and Yang, Y. (2014). Using volcano plots and regularized-chi statistics in genetic association studies. *Computational biology and chemistry, 48*, 77-83. Doi: <https://doi.org/10.1016/j.compbiolchem.2013.02.003>

McCarthy, D. J., Campbell, K. R., Lun, A. T., and Wills, Q. F. (2017). Scater: pre-processing, quality control, normalization and visualization of single-cell RNA-seq data in R. *Bioinformatics, 33*(8), 1179-1186. Doi: <https://doi.org/10.1093/bioinformatics/btw777>

Niedziela, G., Szabelska-Beręsewicz, A., Zyprych-Walczak, J., and Graczyk, M. (2022). Application of edgeR and DESeq2 methods in plant experiments based on RNA-seq technology. *Biometrical Letters*, *59*(2), 127-139. Doi: <https://doi.org/10.2478/bile-2022-0009>
